# Epistemic motivation affects the processing of negative emotional stimuli in interpersonal decisions

**DOI:** 10.3389/fpsyg.2015.01057

**Published:** 2015-07-23

**Authors:** Zhenyu Wei, María Ruz, Zhiying Zhao, Yong Zheng

**Affiliations:** ^1^Key Laboratory of Cognition and Personality, Ministry of Education, School of Psychology, Southwest UniversityChongqing, China; ^2^Mind, Brain, and Behavior Research Center, University of GranadaGranada, Spain; ^3^Key Laboratory for NeuroInformation of Ministry of Education, School of Life Science and Technology, University of Electronic Science and Technology of ChinaChengdu, China

**Keywords:** epistemic motivation, need for cognitive closure, emotional facial displays, interpersonal decision-making, ERP

## Abstract

The present electrophysiological study investigated the role of the need for cognitive closure (NFC) in emotional processing. The NFC is conceptualized as an epistemic motive that is related to how and why people seek out information in social environments. Event-related potentials were recorded while individuals with high NFC (i.e., low epistemic motivation) or low NFC (i.e., high epistemic motivation) performed a modified Ultimatum Game, in which the emotions of happy or angry game agents were employed to predict their most likely offer. High-NFC participants more closely adhered to the decisions rules of the game than low-NFC individuals did. The electrophysiological results showed that the dispositional NFC modified early perceptual components (N170, N200, and P200). The potentials showed that high-NFC subjects had a processing bias to angry faces, whereas low-NFC individuals exhibited no such effects. These findings indicated that high-NFC individuals were more sensitive to negative emotional stimuli than low-NFC individuals in an interpersonal decision-making task.

## Introduction

Previous studies have reported that individuals differ in their dispositional need for cognitive closure (NFC; Kruglanski and Webster, 1996). The NFC is conceptualized as an epistemic motive that is related to how and why people seek out information in their social environments (Calogero et al., 2009) and that varies in individuals according to their motivation for information processing and judgment (Webster and Kruglanski, 1994; Kossowska, 2007; Chirumbolo and Leone, 2008). The NFC is defined as the tendency to reduce experiences of discomfort that are generated by uncertainty and the desire to find a definitive answer to a question (Kruglanski and Webster, 1996). The NFC is a dimension of stable individual disposition (Webster and Kruglanski, 1998; Kruglanski, 2004). High-NFC people have an aversion for ambiguity and therefore low epistemic motivation. These people can be characterized as having preferences for quick decision-making, order, and predictability and as closed-minded (Kruglanski and Webster, 1996). In contrast, low-NFC individuals show a higher tolerance for ambiguity and therefore have high epistemic motivation. They prefer slow decision-making, uncertainty, variety, and openness. The NFC is assumed to instill two general and sequential tendendcies in decision-making: urgency and permanence. The first stage of this process refers to the inclination to quickly determine an answer. High-NFC individuals feel anxious when there is a postponement of the completion of a decision. The second stage preserves the decision by freezing on the acquired structure. The desire of these individuals for permanency lies in the avoidance of their return to uncertainty if the decision is reconsidered. Thus, high-NFC people tend to reject new information after they have reached a decision.

Facial displays of emotions play an essential role in interpersonal interactions. Research on emotional contagion has reported that emotional displays can evoke affective reactions in others (Hatfield et al., 1992). In addition, humans engage in frequent anticipation or prediction of the behavior of others (Frith and Frith, 2007), and facial emotional displays are an important source of these predictions (see, for example, Ekman and Friesen, 2003; Alguacil et al., 2015). Emotional displays help individuals gather information about the emotions, beliefs, and future intentions of others, as well as appraise the current situation, and serve as tools for coordinating interactions (Keltner and Haidt, 1999). The action tendencies of cooperation and competition are thus mainly built on social judgments (Forgas, 1991) and predictions (Frith and Frith, 2007).

Previous investigations have suggested that people with low epistemic motivation directly base their behavior on their affective state that is evoked by the emotional displays of others (Van Kleef et al., 2009). Hence, in the current investigation, we explored the effects of emotional displays during interpersonal decisions in people with high vs. low needs for closure. We employed a modified Ultimatum Game (UG) that was developed by Ruz and Tudela (2011) and that integrates the interpersonal interaction factor with the perception of emotional facial expressions. The UG is often used to investigate interpersonal interactions. In the original game, one player (the *proposer*, who will be named *agent* hereafter) allocates money to himself/herself and to another player (the responder). The responder can either accept or reject the offer. If the responder accepts, both players win their respective amounts, but if the responder rejects the offer, they both receive nothing. The game results show that people reject a high proportion of unfair offers, which does not correspond with a rational perspective (Camerer, 2003).

In the modified UG that was employed in the current study, the participants always played the role of responder. They were required to use the emotions of happiness and anger that were conveyed by the agent to *predict* their likely future offer (good or bad). Some of the agents were trustworthy, while others were untrustworthy. The trustworthy agents’ emotions indicated their natural consequences so that a smiling expression meant that the offer would likely be good, whereas an angry face meant that the offer was most likely bad. However, the untrustworthy agents’ emotions indicated the opposite meanings. The predictions of the cue and the emotional expression of the target were valid in 80% of the trials, which rendered the decision situation uncertain.

We aimed to test two hypotheses with this task. First, because high-NFC individuals have a clear tendency to adhere to norms (Fu et al., 2007), we hypothesized that they would be more influenced by the probabilities of the decision rule or the predictions that were generated by the combination of the cue and the emotional expression of the target, which were valid in 80% of the trials, compared with low-NFC individuals. Second, based on the findings of previous studies (Van Kleef, 2009; Van Kleef et al., 2010), we predicted that people with high-NFC would be more sensitive to displays of anger. Compared with people with high epistemic motivation, individuals with low epistemic motivation are more influenced by the affective reactions that are elicited by the angry expressions of other people (Van Kleef et al., 2009). In our study, we hypothesized that the NFC would influence the electrophysiological processing of negative emotional information when people responded to an agent in interpersonal contexts. We therefore focused our analyses on the potentials that reflect emotional and attentional processing. First, previous studies have shown that the N170 component is modified by negative facial expressions (Blau et al., 2007; Krombholz et al., 2007). In these studies, the N170 amplitudes were larger in response to angry faces compared to happy faces (Blau et al., 2007; Krombholz et al., 2007; see also Tortosa et al., 2013). Second, the results of Campanella et al. (2002) suggest that the N200 component is sensitive to the presentation of negative facial expressions and may signal a change of attention toward biologically significant events. Other studies have also shown that the N200 is sensitive to the presentation of negative facial expressions and other negative emotional information (Eimer and Holmes, 2002; Kanske and Kotz, 2010; Ruz et al., 2012). Third, the P200 potential is associated with the processing of negative emotional stimuli (Schutter et al., 2004). In some studies, this component has been found to be larger for negative than for positive stimuli (Carretié et al., 2001; Delplanque et al., 2004). Fourth, the N300 is a negative deflection that is sensitive to emotional stimuli (Carretié et al., 1997). Previous studies have reported that angry facial expressions generate a larger N300 than happy ones (Schutter et al., 2004; Ruz et al., 2012). Last, several studies have reported a larger late positive potential (LPP) in response to emotional relative to neutral stimuli (e.g., Schupp et al., 2000; Carretié et al., 2001). Such patterns have been interpreted as indicative of the deeper processing of information and greater allocation of attentional resources to emotional stimuli. Hence, we hypothesized that high-NFC individuals would show heightened electrophysiological differences between happy and angry faces in the aforementioned components.

## Materials and Methods

### Participants

Participants were selected from a pool of 111 Chinese undergraduates. All were native Mandarin speakers, with no neurological or psychological disorders, and with (corrected to) normal color vision. Written informed consent was obtained after detailed explanation of the experiment. The study was approved by the Ethics Committee of Southwest University. Participants were divided into two groups on the basis of their score of the Need for Cognitive Closure Scale (NFCS). The ERP experiments included 14 participants in high-NFC (mean age = 21.5, 4 males) and 14 subjects in low-NFC (mean age = 21.7, 1 male)^1^.

### Measures

In our study, NFC was measured with the Chinese version (Liu et al., 2007) of the Webster and Kruglanski (1994) scale. Accurate translation ensured that the Chinese version was consistent with the original version. The scale has a high test–retest reliability (*r* = 0.8611) observed over a 12–13 week period, which indicates that the personality construct it taps is relatively stable (Webster and Kruglanski, 1994). Also, the scale has received confirmation and cross-cultural validation in a series of investigations (Chiu et al., 2000; Moneta and Yip, 2004; Kossowska, 2007). Subjects answered the questionnaire by responding to a 6-point scale ranging from 1 (strongly disagree) to 6 (strongly agree). As the original scale, the Chinese version is a 47-item questionnaire. Subjects’ total score is calculated by summing the scores of 42 items, and the remaining five questions are used to obtain a lie index. Additional analyses indicated that the Chinese version of NFCS also possesses high internal consistency (Cronbach’s *α* = 0.77).

Fourteen participants whose scores were above the 75th percentile of the NFC distribution (score exceeding 172) were classified as high-NFC, and 14 subjects whose scores were below the 25th percentile (score below 142) were classified as low-NFC. All of them participated in the electrophysiological experiment.

### Stimuli and Procedure

Our experiment contained three blocks (forty trials each; 120 trials in total). After the first fixation point (+; 1° × 1°) was presented with a variable 1000–1500 ms duration, the cue (either a triangle or a circle, 2° × 2° approximately) appeared at the center of the screen for 500 ms, followed by a variable inter-stimulus interval displaying another fixation point for 1000–1500 ms. Afterward the face of the agent (5° × 6°) was presented for 500 ms, followed by another fixation point (1° × 1°) lasting between 1000 and 1500 ms. Then, the words “please make a choice” were presented. Participants made their decision by pressing the appropriate button (1 or 2 keys on the keyboard) during the choice time. This time period was limited to 10 s^2^. The offer (3.5° × 1.5°) was presented for 500 ms immediately after subject responded.

The 120 faces were divided into 60 angry (50% female, 50% male) and 60 happy expressions (50% female, 50% male) taken from the Chinese Facial Affective Picture System (CAPS; Wang and Luo, 2005). These faces were presented in random order, and were similar in perceptual intensity (mean: angry = 5.58, happy = 5.66; *t*(59) = 1.129, *p* = 0.263; Chinese Affective Picture System; Wang and Luo, 2005).

**FIGURE 1:**
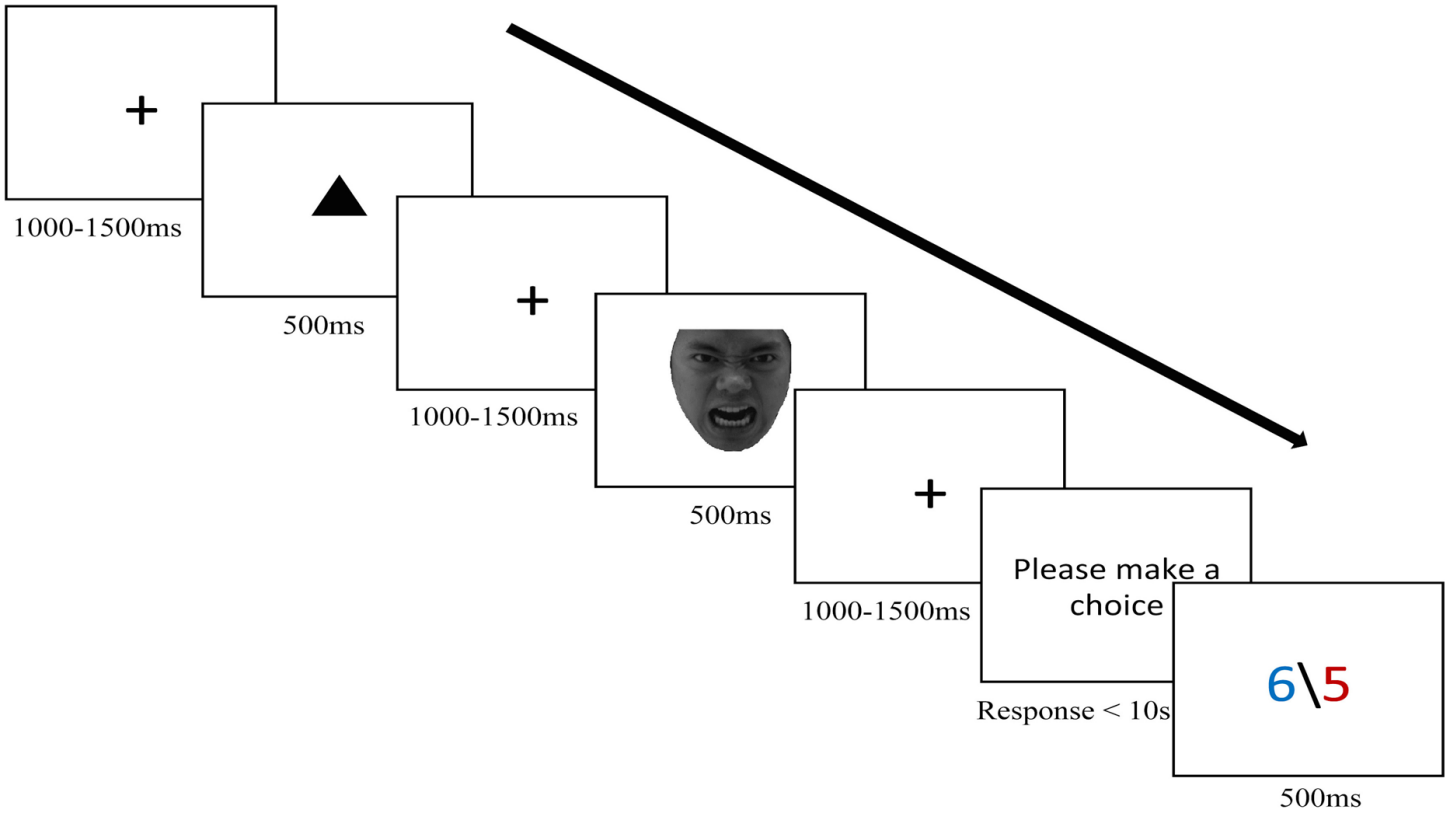
**Demonstration of sequence of events in a trial (take angry face condition for example)**.

Participants received the instruction that the agents in the game had completed a questionnaire related to trustworthiness. Some agents were trustworthy while the others were untrustworthy. Participants could judge how trustworthy the agents were on the basis of the cue presented at the beginning of every trial. If the agent was trustworthy, a circle (triangle) would be presented. Or if the agent was untrustworthy, a triangle (circle) would appear. The trustworthy agent smile implied that the offer was probably good for the participant and an angry expression meant that the offer was probably bad. On the contrary, the untrustworthy agent smile meant that the offer was probably bad, whereas an angry expression indicated that the offer was probably good. The validity of this information was 80% (that is, the situation was uncertain). Thus, offers were good to the subjects in 80% of the trials in which a trustworthy agent smiled or an untrustworthy agent was angry. On the other hand, offers were bad in 80% of the trials in which a trustworthy agent had an angry face or an untrustworthy agent smiled. Participants were asked to use the combined information of the cue and the emotional expression of the agent to predict their most likely offer and either accept or reject it before the actual offer was presented. Once the participant decided, the offer was displayed. If the participant accepted the offer, the money would be divided as proposed and the agent and participant would win their respective amounts. If the participant rejected the offer, instead, none of them obtained anything for that trial. The responder’s goal was to gain more money than the agents. Participants completed a short training session (10 trials) before performing the main experiment. The training task had the same parameters as the main task except the faces (which were another 10 facial photographs). The sequence of events in a trial is illustrated in **Figure 1**.

Extraneous variables in our study were strictly controlled. (1) The meaning of the trustworthiness cue was counterbalanced across participants. (2) The numbers in the offers were taken from 1 to 9 and separated by a slash. The difference between the two numbers was always 1. The color of the number, which was assigned to the participant, was blue (red) and the other number, which was assigned to the agent, was red (blue). The color distribution was counterbalanced across participants. (3) The location and color of the highest number were matched across trials. There were 32 different offers.

### Electrophysiological Recording and Analysis

Electroencephalography (EEG) was recorded from 64 scalp sites using tin electrodes mounted in an elastic cap (Brain Products), with the reference on the left and right mastoids and a ground electrode on the medial frontal aspect. The vertical electrooculogram (EOG) was recorded with electrodes placed above and below the left eye. The horizontal EOG was recorded as the left versus right orbital rim. All electrode impedances were maintained below 5 KΩ. The EEG and EOG were amplified using a 0.05∼100 Hz band pass and continuously digitized at 500 Hz/channel. ERP averages were computed off-line. Eye movement artifacts (eye blinks and movements) were excluded oﬄine. Trials with EOG artifacts (EOG voltage exceeding ±80 μV), amplifier clipping and peak-to-peak deflection exceeding ±80 μV were excluded from averaging. All other trials satisfying these thresholds were included in the averages. The data were baseline-corrected with respect to the pre-stimulus (face) interval of 200 ms. ERP waveforms were time-locked to the onset of the face and the average epoch was 700 ms, including a 200 ms pre-stimulus baseline.

We selected electrode sites for statistical analysis according to previous studies. For the N170, only the PO4 electrode where the most prominent N170 potential was observed in all conditions was analyzed (same site as Ibáñez et al., 2012). For the N200, the following five electrodes were analyzed: FCZ, FC1, FC2, FC3, FC4 (five frontal sites, consistent with Yuan et al., 2007). For the P200, we employed the following electrodes: CPZ, CP1, CP2, CP3, CP4 (five central sites, based on Jessen and Kotz, 2011). And for the N300 and LPP we selected the following electrodes: FZ, F1, F2, F3, F4, and CZ, C1, C2, C3, C4 (five frontal sites and five central sites, based on Schupp et al., 2000; Schutter et al., 2004). The mean amplitudes of the N170 (150–200 ms), N200 (200–250 ms), P200 (250–300 ms), N300 (300–350 ms), and LPP (350–500 ms) components were measured and analyzed.

The average number of trials per condition entered in the electrophysiological analysis was 30. The present study focused on the relationship between NFC and emotional displays, and therefore, we did not add the trustworthiness factor to the electrophysiological analysis to simplify the design. Adding the trustworthiness variable to the electrophysiological ANOVA did not bring any additional insight into the relationship between NFC and emotional processing, and thus we decided not to include it for the sake of clarity. ERP data was analyzed by means of a 2 × 2 × 5 ANOVA with Group as a between-participants factor (high-NFC vs. low-NFC) and Emotion (angry, happy) and five electrode sites as within-participants factors. To analyze the N170, we used a 2 × 2 ANOVA^3^. The degrees of freedom were corrected according to the Greenhouse–Geisser method. Whenever significant effects were revealed in the ANOVA, subsequent ANOVAs and least significant difference (LSD) tests were applied to identify the sources of main effects and interactions.

## Results

### Behavioral Performance

All trials were included in the analysis of reaction times (RTs)^4^. RTs data was analyzed by means of a 2 × 2 × 2 ANOVA with the between group factor Group and Emotion and Trustworthiness as within-subject factors. The ANOVA indicated a main effect of emotion, *F*(1,26) = 13.62, *p* < 0.01, as the RT was longer in the angry (*M*_angry_ = 902.12, SD = 101.02) than in the happy condition (*M*_happy_ = 755.87, SD = 74.10). Responses were slower for untrustworthy (*M* = 918.76, SD = 99.10) than for trustworthy agents (*M* = 739.23, SD = 74.83), *F*(1,26) = 31.62, *p* < 0.001. The interaction between trustworthiness and emotion was also significant, *F*(1,26) = 9.25, *p* < 0.01. The results showed that the difference between the two types of emotion was larger in the trustworthy [*F*(1,26) = 19.02, *p* < 0.001; *M*_angry_ = 850.18, SD = 94.27; *M*_happy_ = 628.29, SD = 60.05] than in the untrustworthy [*F*(1,26) = 2.79, *p* = 0.107; *M*_angry_ = 954.06, SD = 108.79, *M*_happy_ = 883.46, SD = 93.29] condition.

Regarding participants’ choices, we found that high-NFC subjects rejected the offers at a significantly higher rate (12.5%) than low-NFC group (6%) in the trustworthy-happy condition, and accepted the offers at a significantly higher rate (19.6%) than low-NFC individuals (3.9%) in the trustworthy-angry condition (trustworthy-happy: *χ*^2^_(1)_ = 10.86, *p* < 0.01; trustworthy-angry: *χ*^2^_(1)_ = 44.18, *p* < 0.001). These effects were not significant in the untrustworthy situation (untrustworthy-happy: *χ*^2^_(1)_ = 3.32, *p* = 0.069; untrustworthy-angry: *χ*^2^_(1)_ = 1.95, *p* = 0.162).

### Electrophysiological Data

#### N170

**FIGURE 2:**
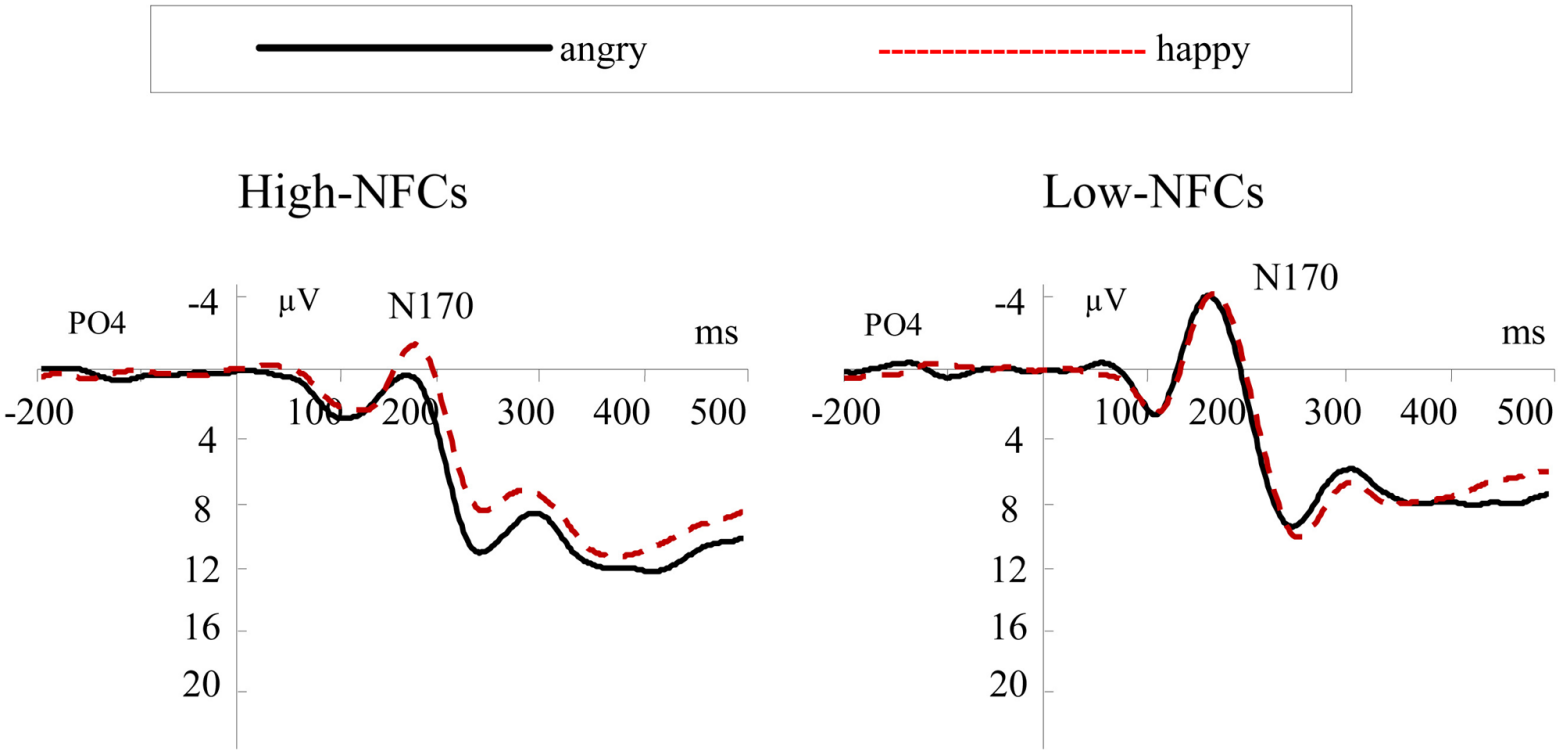
**Grand average ERP waveforms at PO4 for angry (solid lines) and happy (dashed lines) faces for the high-NFC and low-NFC groups**.

As shown in **Figure 2**, the N170 component peaked at 170 ms. The ANOVA indicated a significant main effect of emotion, *F*(1,26) = 21.51, *p* < 0.001. The amplitude of this potential was larger for happy (*M* = -2.06; SD = 1.82) than for angry faces (*M* = -0.79; SD = 1.80). We also found a significant interaction involving Group and Emotion, *F*(1,26) = 6.09, *p* < 0.05. The results showed that the N170 of high-NFC participants was more positive for angry compared to happy faces, *F*(1,26) = 25.25, *p* < 0.001. This effect was absent in the low-NFC, *F*(1,26) = 2.35, *p* = 0.137 (for details about amplitudes see **Table 1**).

**Table 1:** Mean N170, N200, and P200 amplitudes (*M*) and SD for the angry and happy faces in each group.

Measure	High-NFC	Low-NFC
N170		
Angry	1.167 (7.828)	-2.743 (10.983)
Happy	-0.783 (7.487)**	-3.339 (11.332)
N200		
Angry	2.864 (4.069)	4.524 (7.255)
Happy	1.118 (3.524)w	4.022 (6.814)
P200		
Angry	5.885 (6.03)	8.222 (7.91)
Happy	4.267 (5.387)*	8.479 (7.838)

*p < 0.05, **p < 0.001.

#### N200

**FIGURE 3:**
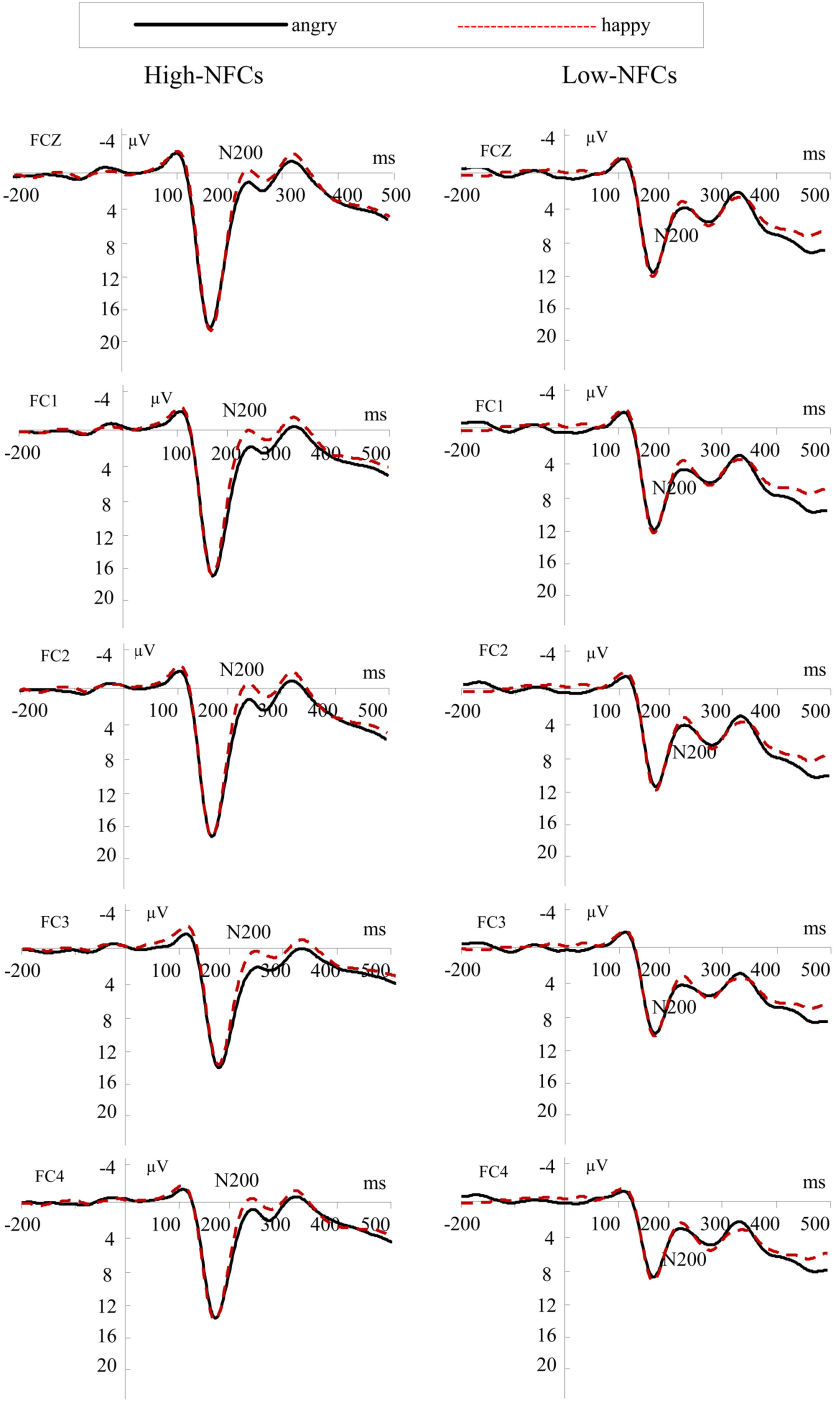
**Grand average ERP waveforms at FCZ, FC1, FC2, FC3, FC4 for angry (solid lines) and happy (dashed lines) faces for the high-NFC and low-NFC group**.

**Figure 3** shows grand-averaged ERPs in response to different emotional faces. The N200 peaked at a mean latency of 240 ms. The ANOVA conducted on N200 amplitudes yielded a significant main effect of Emotion, *F*(1,26) = 14.24, *p* < 0.01. Angry faces elicited more positive amplitudes (*M* = 3.69; SD = 1.13) than happy faces (*M* = 2.57; SD = 1.04). There was also a significant interaction between Group and Emotion, *F*(1,26) = 4.36, *p* < 0.05. Individuals with high-NFC displayed an N200 of more positive amplitude to the angry compared to the happy face, *F*(1,26) = 17.18, *p* < 0.001, whereas the N200 of low-NFC subjects showed no amplitude difference between the two conditions [*F*(1,26) = 1.42, *p* = 0.244; for details about amplitudes see **Table 1**].

#### P200

**FIGURE 4:**
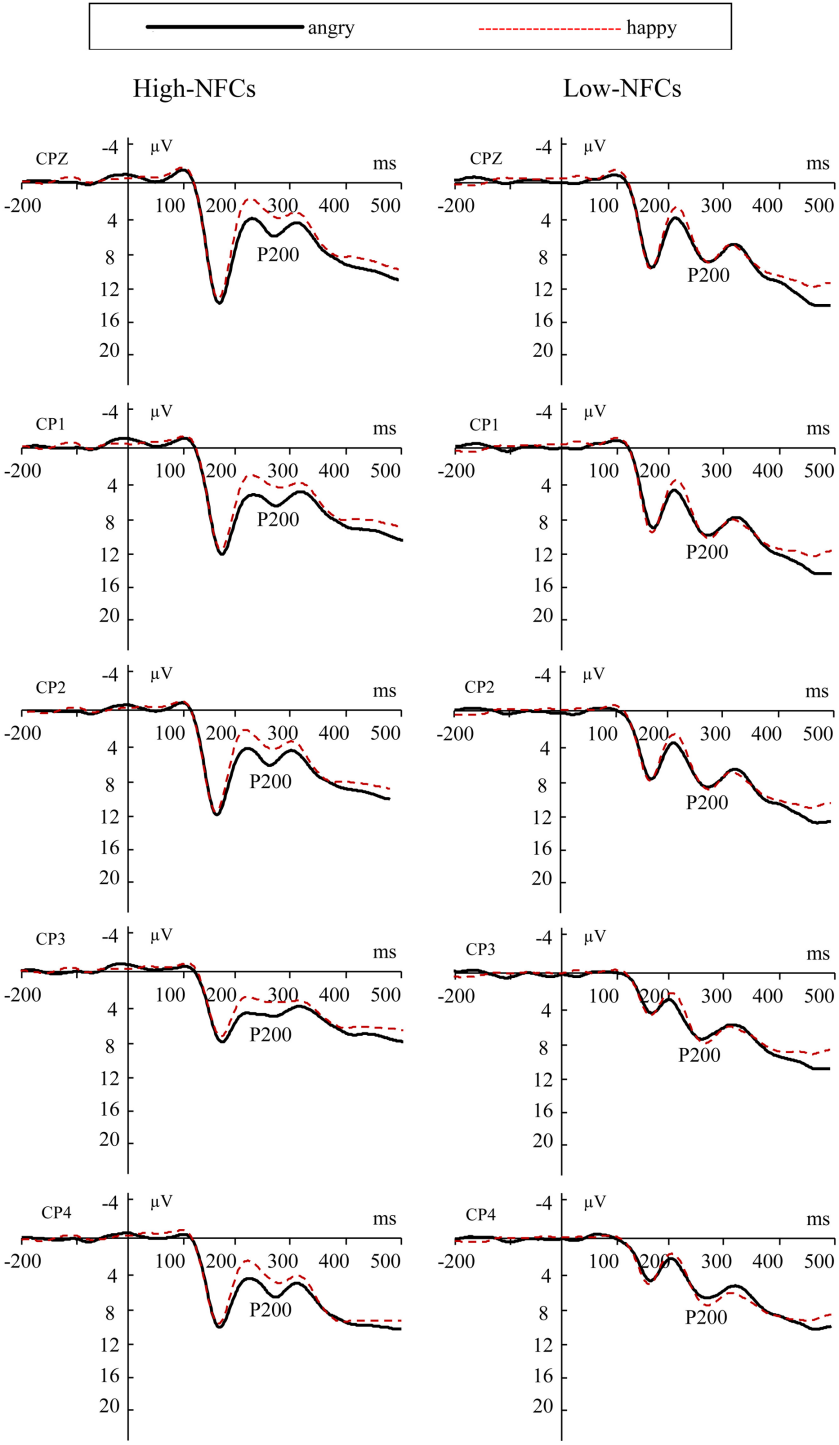
**Grand average ERP waveforms at CPZ, CP1, CP2, CP3, CP4 for angry (solid lines) and happy (dashed lines) faces for the high-NFC and low-NFC group**.

**Figure 4** displays the P200, which peaked at a mean latency of 270 ms. The ANOVA on the averaged amplitudes of this potential showed that there was no main effect of Emotion [*F*(1,26) = 2.33, *p* = 1.139]. The interaction between Emotion and Group was significant, *F*(1,26) = 4.43, *p* < 0.05. In the same line as before, the P200 of high-NFC participants displayed enhanced amplitudes to angry faces compared to happy ones, *F*(1,26) = 6.59, *p* < 0.05. However, low-NFC participants showed no difference in P200 amplitudes in the two conditions (*F* < 1; for details about amplitudes see **Table 1**).

#### N300

N300 amplitudes showed no significant main effect of Emotion (*F* < 1). The interaction between Emotion and Group was not significant either (*F* < 1).

#### Late Positive Potential

Late positive potential amplitudes showed no significant main effect of Emotion, *F*(1, 26) = 3.416, *p* = 0.076 (*M*_happy_ = 8.67, SD = 1.232; *M*_angry_ = 9.17, SD = 1.273). The interaction between Emotion and Group was not significant either, *F*(1,26) = 3.738, *p* = 0.064.

## Discussion

In the present study, we employed an economic game in which agents displayed emotional faces to examine how high- and low-NFC individuals process emotional facial displays in interpersonal contexts. Our behavioral results showed that participants took longer to respond to angry compared to happy faces and that they were slower to respond to untrustworthy than to trustworthy agents, regardless of their NFC psychological disposition. The difference in the responses to angry faces and happy faces was greater in the trustworthy condition compared with the untrustworthy condition. These behavioral results were consistent with the results of the study by Ruz and Tudela (2011). Previous studies have suggested that increased attentional resources are devoted to negative events because negative information may be a symbol of a potential danger and that this difference was evolutionally acquired (Carretié et al., 2001; Campanella et al., 2002; Wang et al., 2011). However, it is also common to observe slower responses to displays of anger, and this could possibly be related to the avoidance tendencies that they elicit (Marsh et al., 2005).

In the analysis of the frequency of choices (accept or reject) in the trustworthy situation, we found that high-NFC participants were more influenced by the probabilities of the decision rule (that is, the information about the most likely offer that was provided by the combination of the cue and facial expression information, which was valid on 80% of the trials) than low-NFC subjects were. The rate of acceptance of the high-NFC participants matched the probabilities of the feedback more closely than the decisions of the low-NFC individuals. When the agents were trustworthy and happy, high-NFC individuals chose to reject the offer at a significantly higher rate than the low-NFC group did. In addition, when the agents were trustworthy and angry, high-NFC individuals chose to accept the offer at a significantly higher rate than low-NFC individuals did.

In the electrophysiological analyses, we found that high-NFC individuals showed heightened differences between facial emotional expressions, which were evidenced by differences in the N170, N200, and P200 amplitudes for angry vs. happy agents, and these differences were not observed in low-NFC participants.

### N170

The N170 component is a reliable index of the initial stages of facial feature coding (Blau et al., 2007; Wronka and Walentowska, 2011). Negative emotions tend to generate larger N170 amplitudes compared to positive ones (Krombholz et al., 2007). However, some previous studies failed to find this effect (Eimer et al., 2003; Holmes et al., 2003). A number of studies have claimed that the N170 component reflects the early structural processing of faces and that it is unaffected by emotional expression (Bentin et al., 1996; Eimer, 2000). The results of our study suggested that the N170 component reflected the emotion that was displayed by the agents’ face. However, participants in the high-NFC group showed a reverse pattern in that the N170 component exhibited smaller amplitudes in response to angry versus happy faces. The amplitude of a positive component may indicate the amount of processing resources that subjects allocate to a task, and studies have found that negative components sometimes display a reversal in this pattern (Mecklinger et al., 1992). In fact, Aranda et al. (2010) found that the N170 component displays an inverted pattern for the validity of faces, in which validly cued faces resulted in less amplitude than invalid faces, and attentional enhancements were observed in the RTs. Therefore, the observed pattern may indicate that high-NFC individuals are more sensitive to negative emotional information (angry faces) in contrast to low-NFC subjects during interpersonal decisions.

### N200

Previous studies have shown that the N200 component is sensitive to the presentation of negative facial expressions and negative emotional information (Campanella et al., 2002; Eimer and Holmes, 2002; Kanske and Kotz, 2010; Ruz et al., 2012). In the present study, high-NFC individuals showed smaller N200 amplitudes in response to negative faces compared to positive ones. This phenomenon was consistent with the results reported by Pérez-Edgar and Fox (2003). Previous studies have reported that, compared with people with high epistemic motivation, individuals with low epistemic motivation may be more influenced by the affective reactions that are elicited by the angry expressions of other people (Van Kleef et al., 2009). Combined with this finding, the N200 results suggested that high-NFC people were more sensitive to negative faces than positive emotional faces in interpersonal decisions. These results suggest that high-NFC individuals easily exhibit negative affective reactions in response to others’ anger.

### P200

The P200 component may index the evaluation and processing of emotional information, and it provides evidence that the emotional modulation of attention happens extremely rapidly in response to socially and emotionally salient stimuli, such as faces (Dennis and Chen, 2007). The P200 component may also be an index of negative evaluations (Carretié et al., 2001; Yuan et al., 2007), threat-related attentional biases (Bar-Haim et al., 2005), negative biases (Huang and Luo, 2006), the detection of extremely dangerous features (Correll et al., 2006), and cognitive resource occupancy (Fabiani et al., 2000). Our results in the P200 component revealed a bias toward the enhanced processing of angry expressions in the high-NFC subjects but not in the low-NFC subjects, which was shown by the enhancement of this potential in response to angry faces in the former group. This effect may be a threat-related attentional bias that is associated with the relationship between NFC and anxiety. Previous research reports that cognitive closure is associated with an insecure attachment style (Mikulincer, 1997). Individuals with insecure attachment are more readily threatened by information that challenges their knowledge structures, especially when they are being emotionally overwhelmed (Mikulincer, 1997). They may anxiously hold on to their initial knowledge constructions when they experience emotional dysregulation (Fonagy and Allison, 2014). In addition, Roets and Van Hiel (2008) found that decision-making acts as a stressor for high NFC individuals. The increased levels of negative arousal in response to anxiety or stress that occur in these individuals could explain why they are more sensitive to negative emotional information during interpersonal decision-making.

### N300 and LPP

It is noteworthy that there were no significant interactions between the NFC and the valence of emotions in the N300 and LPP components, which are the later components. This phenomenon suggested that the NFC affects only the earlier processing stages of facial emotional processing. The later stages appear to be independent of this dispositional factor.

Several limitations of this study should be noted. First, we did not control for the participants’ intelligence levels nor their socio-economic status. Future studies should control these factors, and this will make the results more valid. Second, the level of uncertainty was not manipulated in the present study. Thus, we could not compare the observed effects in high- vs. low-certainty conditions. This manipulation is needed in a future study. Additional studies can use this approach to investigate whether an uncertainty in context arouses feelings of negative emotional experiences in high-NFC individuals. In addition, the mixed effects in previous literature regarding the modulation of emotional valence on ERP amplitudes show that this phenomenon is not fully understood. In this sense, the current results only represent a step forward providing additional evidence to the field.

Despite these limitations, the present study suggests that high-NFC individuals have a negative bias toward processing anger in interpersonal decisions. The high-NFC subjects showed a processing bias to angry faces compared to happy ones, which was evidenced in the N170∖N200∖P200 potentials. Van Kleef et al. (2009) found that people with low epistemic motivation exhibit negative affective reactions in response to others’ anger. Our findings were in agreement with this, and these results have implications for theories on emotional contagion (Van Kleef, 2009; Van Kleef et al., 2009). The results reported here add electrophysiological evidence to the results of previous studies that have shown that individuals with low epistemic motivation exhibit heightened affective reactions in response to emotional faces (Van Kleef et al., 2009, 2010) and suggest that these reactions could be mediated by the attentional enhancement of emotional processing.

## Conflict of Interest Statement

The authors declare that the research was conducted in the absence of any commercial or financial relationships that could be construed as a potential conflict of interest.

## Footnotes

1 Although groups did not have a matching number of female and male participants, previous studies suggest that gender does not affect individuals’ score on the NFCS (Webster and Kruglanski, 1994).

2 We gave plenty of time to subjects to decide with the goal of avoiding a potential influence of time pressure on the need for cognitive closure (NFC). The following slide appeared immediately after participants responded.

3 The analysis of the N170 is a 2 × 2 ANOVA because the number of the electrode is only one.

4 There are no trials in which no responses occurred.
